# Phototracking Vaccinia Virus Transport Reveals Dynamics of Cytoplasmic Dispersal and a Requirement for A36R and F12L for Exit from the Site of Wrapping

**DOI:** 10.3390/v10080390

**Published:** 2018-07-24

**Authors:** Helena Lynn, Liam M. Howell, Russell J. Diefenbach, Timothy P. Newsome

**Affiliations:** 1 School of Life and Environmental Sciences, The University of Sydney, Sydney, NSW 2006, Australia; helena.lynn@sydney.edu.au (H.L.), lhow8488@uni.sydney.edu.au (L.M.H.); 2 Department of Biomedical Science, Faculty of Medicine and Health Sciences, Macquarie University, Sydney, NSW 2109, Australia; russell.diefenbach@mq.edu.au

**Keywords:** vaccinia virus, transport, microtubule, kinesin, cytoskeleton, photoconvertible fluorescent proteins

## Abstract

The microtubule cytoskeleton is a primary organizer of viral infections for delivering virus particles to their sites of replication, establishing and maintaining subcellular compartments where distinct steps of viral morphogenesis take place, and ultimately dispersing viral progeny. One of the best characterized examples of virus motility is the anterograde transport of the wrapped virus form of vaccinia virus (VACV) from the *trans*-Golgi network (TGN) to the cell periphery by kinesin-1. Yet many aspects of this transport event are elusive due to the speed of motility and the challenges of imaging this stage at high resolution over extended time periods. We have established a novel imaging technology to track virus transport that uses photoconvertible fluorescent recombinant viruses to track subsets of virus particles from their site of origin and determine their destination. Here we image virus exit from the TGN and their rate of egress to the cell periphery. We demonstrate a role for kinesin-1 engagement in regulating virus exit from the TGN by removing A36 and F12 function, critical viral mediators of kinesin-1 recruitment to virus particles. Phototracking viral particles and components during infection is a powerful new imaging approach to elucidate mechanisms of virus replication.

## 1. Introduction

Interactions with the microtubule cytoskeleton and its associated motor proteins mediate subcellular transport of virus particles and anchor viral organelles within host cells [1]. Tracking the subcellular motility of virus particles is a considerable technical challenge for live-cell imaging as they are relatively small objects and therefore require high-powered lenses to resolve them [2]. As they move at very fast rates (maximal velocities that exceed 5 μm/s) and cells may contain many hundreds of virus particles, high-frame rates are needed to track the paths of single virus particles. Both of these factors contribute to the bleaching of samples, eliciting photodamage, and limiting long-term imaging of virus transport. Many of these limitations can be circumvented with the use of photoconvertible fluorescent proteins (PAFPs) such as PS-CFP2, Eos and Dendra2 [3,4,5]. These proteins enable labeled subcellular structures to be photoconverted by exposure to a wavelength that modifies the excitation and emission spectral characteristics of the PAFP within a region of interest. This approach has been used successfully to track subcellular organelles and protein trafficking within live cells, but has not, until now, been used to study virus transport [6,7].

Vaccinia virus (VACV), the prototypic member of the orthopoxvirus genus and live vaccine used in the successful eradication of smallpox, engages with the microtubule network at multiple stages of its replication cycle [8]. Following infection of a susceptible cell, VACV establishes a replication and morphogenesis compartment (the virus factory) at a perinuclear site. As the virus factory matures, infectious Mature Virus (MV) are generated, a subset of which are translocated to the early endosome/*trans*-Golgi network (hereafter referred to as TGN for brevity) [9]. Movement of MV between the virus factory and TGN is also microtubule-dependent and involves the surface viral protein A27 [10,11]. Here, MV are wrapped in two additional membranes, giving rise to Wrapped Virus (WV), a second, infectious morphological variant distinguishable from MV by the acquisition of a complement of integral and membrane-associated WV proteins [12].

Transport from the TGN to the cell periphery is the best-characterised microtubule-dependent transport event during VACV replication and is mediated by kinesin-1, which is recruited to the surface of WV by the combined action of three WV proteins [8]. The Type Ib transmembrane protein A36 engages the tetratricopeptide repeats (TPR) of kinesin light chain (KLC, a component of kinesin-1) via two WE/WD motifs [13,14,15,16]. Additionally, a complex of F12 and E2 binds to KLC (E2 interacts with the KLC-2 isoform) [17,18]. How F12 and E2 associate with the outer membrane is unclear, although F12 has been shown to interact with A36 in a yeast 2-hybrid assay [19]. Loss of A36 or F12/E2 leads to a profound reduction in the anterograde egress of WV, suggesting that the action of both A36 and the F12/E2 complex are important in this transport step [13,16,17,20,21]. Live-cell microscopy of fluorescent viruses has revealed that WV move in a saltatory manner with average speeds of 45 μm/min, however peak velocities of 90 μm/min have been observed [20]. At the cell periphery, exocytosis of WV is followed by the initiation of actin nucleation in the underlying cytoplasm by extracellular virus to promote the cell-to-cell spread of infection [22].

For this study, we have generated recombinant VACV carrying Dendra2 fusions to WV and MV localized viral proteins: VACV B5R-Dendra2, VACV F13L-Dendra2 (both expressed at TGN and on WV) and VACV Dendra2-A3L (expressed at virus factory, TGN, and on MV and WV). These viruses completed the normal stages in a replication cycle with some delay in replication kinetics particular to some strains evident in plaque assays. Fluorescent viruses were able to be efficiently photoconverted by brief exposure to light with a wavelength of 405 nm and tracked over 30 min intervals. Through photoconversion of the TGN, we were able to demonstrate the egress of 31.1 ± 3.92 WV particles to the cytoplasm at 15 min post-conversion. Disrupting kinesin-1 transport by introducing null mutations in A36 or F12 led to 2-fold reduction of WV that had exited the TGN, and those that did exit failed to disperse to the cell periphery at the same rate as the parental strain. We could also show that the origin of WV within the TGN during wrapping is a strong predictor for the subcellular address that the progeny virus are fated to track to. Our results validate the use of PAFP-expressing recombinant VACV to examine aspects of virus transport previously opaque to standard imaging studies and support a role for kinesin-1 engagement in regulating the exit of WV from the TGN.

## 2. Materials and Methods

### 2.1. Plasmid Constructs

We constructed a recombination plasmid to generate a recombinant virus that expresses a Dendra2-A3L fusion protein from the endogenous A3L locus, referred to as pDendra2-A3L. We used a two-step fusion PCR strategy to construct pDendra2-A3L. The left arm of A3L was amplified from VACV WR (ATCC VR-1354) genomic DNA with the primer pair a3_5/a3_6 and the Dendra2 gene was amplified from the Dendra2-N vector (Evrogen (FP822), Moscow, Russia, kind gift from K. Lukyanov) with a3_7/den_1 primer pair. The second step of the fusion PCR utilised these first step products as the template with a3_5/den_1 to generate a full-length product. This LA-Dendra2 fragment was then cloned with the restriction endonucleases SalI and HindIII into a GPT selection vector which contained an *Escherichia coli gpt* gene that encodes guanine-hypoxanthine phosphoribosyltransferase (GPT, [23] and screenable marker gene monomeric Red Fluorescent Protein (mRFP, [24]). GPT acts as a selection marker by allowing growth of VACV in the presence of mycophenolic acid (MPA; an inhibitor of purine metabolism), xanthine and hypoxanthine [25]. 

To generate B5R-Dendra2 VACV, we constructed pB5R-Dendra2. The Dendra2 gene was amplified from the Dendra2-N vector with den_3/den_4 primer pair and cloned into a pE/L vector [26,27]. The Dendra2 open reading frame was flanked with the B5R ORF (stop codon deleted) and 3’ genomic sequence, both from the previously published B5R-YFP expression construct [17]. A slightly different modular approach was used for construction of pF13L-Dendra2. Using the F13L targeting vector [28], the GFP open reading frame was replaced with Dendra2 following the amplification of Dendra2-N vector with primer pair den_3/den_4 (see Table 1).

### 2.2. Infection

The VACV WR strain (ATCC VR-1354) was a gift from Michael Way, CRICK, and was used as the parental strain for all VACV lines used in or generated in this study. For all infections, growth medium was removed from cells and the cells washed once in PBS. Virus at a specified MOI was diluted in DMEM not supplemented with FBS (serum free medium; SFM) and applied to washed cells. Cells were incubated at 37 °C with 5% CO_2_ atmosphere for 1 h before SFM with virus was aspirated and the cells recovered with fresh growth medium appropriate for the cell line.

### 2.3. Plaque Assays

Confluent BSC-1 monolayers were infected as described above, but recovered with overlaid GIBCO Modified Eagle Medium (MEM; Invitrogen, Carlsbad, CA, USA) supplemented with 292 μg/mL l-Glutamine, 100 Units/mL penicillin, 100 μg/mL streptomycin and 0.45% UltraPure Agarose (Invitrogen) for purification or 1.5% carboxy-methyl cellulose (CMC) for purification or plaque visualisation/staining. Plaques were allowed to form for 3–5 days before examination.

If plaque assays were for purification, plaques were picked by using a P1000 pipette tip to collect an agarose plug and the infected cells immediately beneath the plug. These were resuspended in SFM, frozen and thawed for three cycles before being placed onto a confluent monolayer of BSC-1 in a well of a 24-well plate for amplification. One hour post infection (hpi), an equal volume of DMEM with 10% FBS was added to the well for a final FBS concentration of 5%.

For plaque assays to visualise plaque morphologies, the MEM/CMC overlay was aspirated and cells were washed three times with PBS then stained with crystal violet (0.5% (*w*/*v*) in 20% methanol solution) for 10 min and then washed again three times with PBS. Individual plaques are formed by the lysis or cytopathic effects caused by viral infection and generate visible clearings in the stained cell monolayer. Stained monolayers were scanned with a high-resolution gel scanner (BioRad GS-800, Hercules, CA, USA). Plaque size was measured in pixels using ImageJ (ver. 1.4.4, a public domain imaging processing program developed by the National Institutes of Health [NIH]) to draw a line across the diameter of the plaque (always horizontally) and then converted to mm with a ruler placed for scale.

### 2.4. Fluorescent Virus Generation

B5R-Dendra2 recombinant VACV was generated by the rescue of ∆B5R [29] with the pB5R-Dendra2 plasmid. pB5R-Dendra2 was introduced into cells infected with ∆B5R through Lipofectamine 2000 (Invitrogen) transfection according to the manufacturer’s instructions. Homologous recombination was allowed to occur between genomic DNA and vector for 24 hpi before the cells were scraped into SFM and virus was released from cells by freeze-thawing 3 times in liquid nitrogen. Plaque assays were performed to select for successful recombinants that displayed both fluorescent markers and restored plaque size (the ∆B5R mutant has an attenuated small plaque phenotype [30]. Dendra2 displayed green fluorescence and was imaged with a FITC filter. Three rounds of plaque assays were performed to purify the recombinant virus.

For generation of Dendra2-A3L and F13L-Dendra2 recombinant VACV, recombinations were performed as described above using pDendra2-A3L and pF13L-Dendra2 plasmids, then plaque assays were carried out with the addition of GPT selection medium (25 μg/mL mycophenolic acid [MPA], 250 μg/mL xanthine, 15 μg/mL hypoxanthine) in the overlay. Plaques able to grow under GPT selection that displayed red fluorescence imaged with the TxRed filter of an Olympus BX51 Microscope were picked and purified three times by plaque assay with GPT selection throughout. These intermediate viruses were then allowed to intra-genomically recombine in a GPT selection negative infection, producing viruses which do not encode selection genes [28]. 24 hpi virus was collected and a plaque assay was performed to isolate the desired fluorescent recombinant which displayed green fluorescence imaged with a FITC filter. Two additional rounds of plaque purification were performed without GPT selection before the integrity of viruses was checked with PCR and immunoblot.

To generate B5R-Dendra2/∆F12L and F13L-Dendra2/∆A36R, BSC-1 cells were co-infected with B5R-Dendra and ∆F12L [31], and F13L-Dendra2 and ∆A36R [32] respectively. 24 hpi, cells were lysed and the first of three plaque assays was conducted to select both phenotypes/markers in single plaques. For both B5R-Dendra2/∆F12L and F13L-Dendra2/∆A36R, small plaques compared to a B5R-Dendra2 or F13L-Dendra2 control that also displayed green fluorescence as imaged with a FITC filter were picked. 

### 2.5. Validating B5R-Dendra2/∆F12L and F13L-Dendra2/∆A36R Null Mutants

Pure preparations of B5R-Dendra2/∆F12L and F13L-Dendra2/∆A36R viruses were used to infect a monolayer of BSC-1 cells. After 48 h the cells were scraped and viral genomic DNA isolated by phenol-chloroform extraction as described [33]. Primer pairs that flank the A36R or F12L allele were designed and used to amplify genomic regions (see Table 2). The resulting PCR products were separated by gel electrophoresis in 1.2% agarose gel.

### 2.6. Immunofluorescence Assays

HeLa cells were seeded on glass coverslips, infected with appropriate viruses and fixed with 3% paraformaldehyde (PFA) in cytoskeletal buffer (CB) for 10 min at room temperature before being washed 3 times in PBS and stored at 4 °C.

Before staining, cells were either permeabilized with 0.1% Triton X-100 in CB for 5 min or not permeabilized, as specified. After washing three times in PBS, cells were blocked in blocking buffer for 20 min then incubated for 40 min with suitable primary antibodies diluted in blocking buffer. After an additional three washes with PBS, secondary antibodies diluted in blocking buffer were applied to cells for 20 min. When appropriate, Alexa Phalloidin conjugates were also added to blocking buffer to incubate with secondary antibodies. Coverslips were then washed in MQ water before being mounted on a glass slide with 0.3–1% (*w*/*v*) P-phenylenediamine (Sigma-Aldrich, St. Louis, MO, USA) in mowiol mounting media. Slides were incubated at 37 °C for 30 min until the mowiol mounting media solidified prior to imaging.

IFAs were imaged on Olympus BX51 Microscope with Reflection Fluorescence System (Mercury Burner (U-RFL-T), F-view monochrome fluorescence camera and DAPI (347 nm/442 nm [#31013v2]), eCFP (436 nm/480 nm [#49001]), FITC (495 nm/515 nm [#31001]) and TxRed (584 nm/610 nm [#31004]) Chroma filters) or Nikon Eclipse Ti-E inverted microscope system (Melville, NY, USA), equipped with an Andor Ultra 888 EMCCD camera, a Lumencor Spectra X fluorescent light source, and DAPI (LED-DAPI-A), FITC (LED-FITC-A) and TxRed (TxRed-4040C) filter sets. Micrographs were captured using AnalySIS LS Starter (Olympus Soft Imaging Systems, ver. 2.8, Tokyo, Japan) or NIS-Elements AR (ver. 4.51.01, Tokyo, Japan) respectively, and edited using Photoshop CS5.1 (Adobe, ver. 12.1, San Jose, CA, USA) and ImageJ (NIH, ver. 1.4.4, Bethesda, MD, USA). 

### 2.7. Confocal Microscopy

HeLa cells seeded onto fibronectin coated No. 1.5 glass µ-Dishes (Ibidi) were infected with the appropriate virus and then imaged on an Olympus FV1000 confocal microscope at the Australian Centre for Microscopy and Microanalysis (ACMM, Sydney, Australia). Infected cells were imaged sequentially with a UPLSAPO 60× oil objective lens with 488 nm multi-line argon laser and 559 nm diode laser with 4.0 pixel/s scan speed to obtain pre-activation images. A 40 × 40 pixel region of interest (ROI), the region of the cell with the brightest 488 nm (Dendra2) emission, was selected for photoconversion. The ROI was activated with 3% of the 405 nm SIM laser under “Tornado” setting for 100 ms at a scan speed of 100 µs/pixel. After activation, either a time course or z-stack from the basal to apical surface was collected (step size was determined by automated optimisation).

## 3. Results

### 3.1. Construction of Photoconvertible VACV

**Figure 1:**
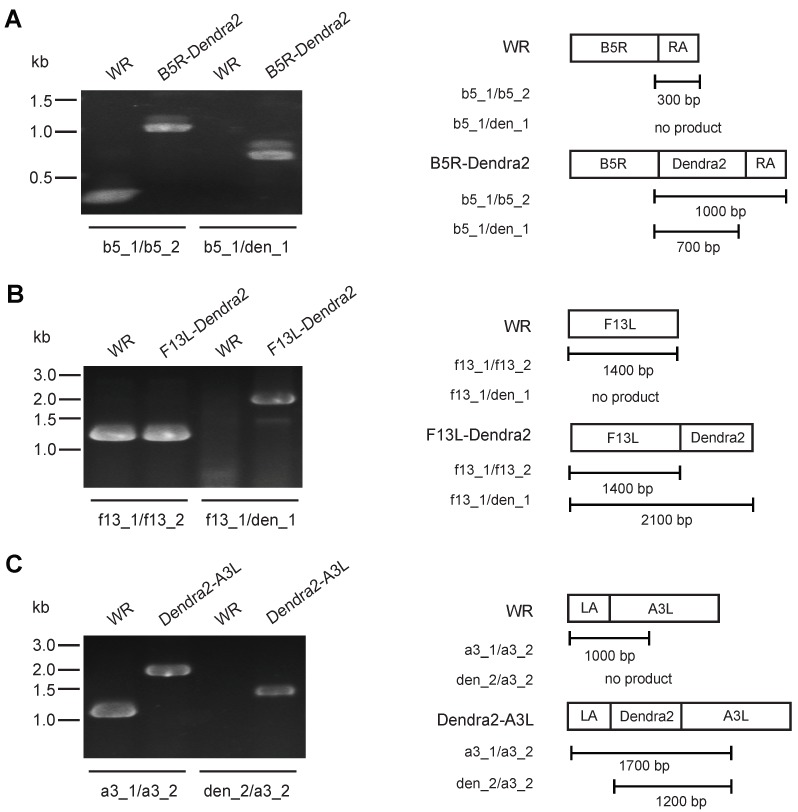
Construction of recombinant VACV viruses expressing Dendra2. (**A**–**C**) Genomic fusions of the Dendra2 open reading frame to B5R, F13L and A3L was confirmed by PCR. Schematic genome representations of gene fusions and expected PCR product sizes from indicated primer pairs are shown on the right.

The complex morphogenesis of VACV leads to the production of two mature infectious forms that localise to the cytoplasm of infected cells, MV and WV. These morphological variants can be distinguished as WV possess two additional membranes, which have a distinct origin and protein complement to the inner membrane that is common to both forms. Tagging WV membrane-specific proteins exclusively labels WV, while tagging core proteins labels both WV and MV. For this reason, we generated recombinant viruses from the prototypal Western Reserve (WR) VACV strain in which the B5R and F13L genes (which encode WV envelope proteins B5 and F13) were fused in-frame to the Dendra2 open reading frame. Dendra2 is derived from the mutagenesis of a fluorescent protein present in the coral *Dendranephthya* spp. (in the class Anthozoa) [5]. This protein exhibits a pre-conversion profile of 490 nm (Ex^max^) and 507 nm (Em^max^) and, following photoconversion by near 400 nm light, a post-conversion profile of 553 nm (Ex^max^) and 573 nm (Em^max^). B5 and F13 proteins have previously been successfully targeted for tagging with fluorescent proteins and have been shown to generate viable viruses that produce bright viruses amenable to live-cell imaging [34]. We also tagged the A3, which is a core MV protein, as A3 is abundant in virus particles and when fused to fluorescent proteins leads to very bright virus particles [35]. Recombination plasmids were constructed and B5R-Dendra2, F13L-Dendra2 and Dendra2-A3L viruses were generated by homologous recombination and validated by PCR of genomic DNA (Figure 1A–C). 

**Figure 2:**
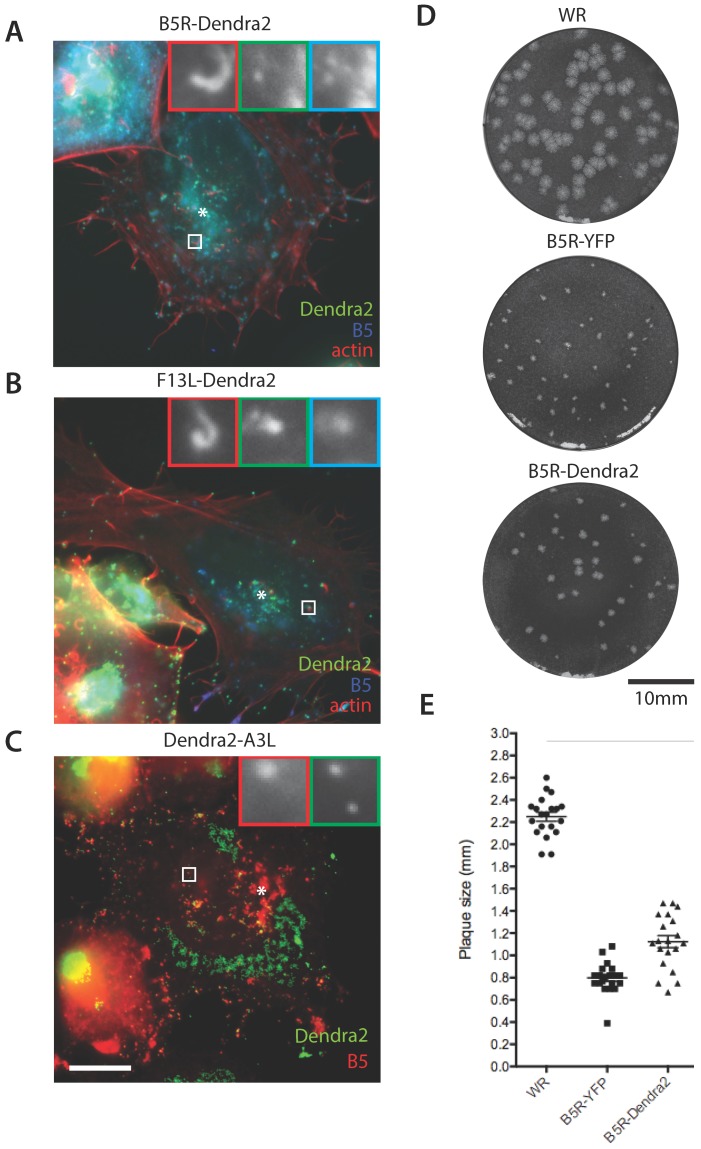
Localization of Dendra2 fusion proteins and plaque assay analysis of Dendra2 tagged viruses. (**A**–**C**) Representative micrographs of HeLa cells infected with indicated viruses at 8 hpi. (**A**,**B**) B5R-Dendra2 (green) and F13L-Dendra2 (green) infected cells were stained with α-B5 (blue) and phalloidin (red). (**C**) Dendra2-A3L (green) infected cells were stained with α-B5 (red). Inserts show single channels of boxed areas. The site of WV wrapping is denoted by an asterisk (*). Scale bar is 10 μm. (**D**) Images of plaque assays performed in BSC-1 cell monolayers with WR, B5R-YFP and B5R-Dendra2 viruses at 3 days post infection and visualized with crystal violet. (**E**) Plaque size for all viruses was quantified as the diameter of the widest horizontal zone of clearing (*n* = 30). All viruses produced statistically different plaque sizes (*p* < 0.0001, Unpaired *t*-test).

To confirm that our photoconvertible recombinant viruses expressed correctly localized fusion proteins we performed immunofluorescence analysis on infected cells (Figure 2A–C). When fused to Dendra2, B5 and F13 were highly expressed at a region adjacent to the nucleus, the site of WV wrapping and at individual virus particles dispersed throughout the cytoplasm. These included viruses that associated with actin comets (determined by colocalisation of phalloidin and anti-B5 antibody, Figure 2A,B) and were therefore determined to be undergoing actin-based motility. In both B5R-Dendra2 and F13L-Dendra2 viruses, Dendra2 localization demonstrated a high level of colocalisation with anti-B5 immunoreactivity as was expected for a WV-tag. Dendra2-A3 protein also localized to virus particles within the cytoplasm but, as expected for a core marker, only a subset of these were positive for B5.

We next performed plaque assays (Figure 2D,E) to test the suitability of Dendra2 as a genetically-encoded fluorescent tag and the extent to which it is functionally neutral. We compared plaques formed by B5R-Dendra2 and the widely used B5R-YFP [17] with the parental VACV WR strain. We determined that B5R-Dendra2 displayed a significantly larger plaque size than B5R-YFP, which, although reduced compared to VACV WR, suggests that Dendra2 may be a superior fluorescent label in this context.

### 3.2. Efficient Photoconversion of Dendra2 Viruses

Having validated the photoconvertible VACV strains, we proceeded to image live cells infected with these viruses on an Olympus FV1000 confocal microscope and test the efficiency of photoconversion. Pre- and post-conversion states of Dendra2 were imaged sequentially with 488 nm and 559 nm lasers, respectively, and photoconversion was performed with a 405 nm laser within a region of interest with conditions optimised to reduce bleaching and maximise photoconversion (see Methods).

**Figure 3:**
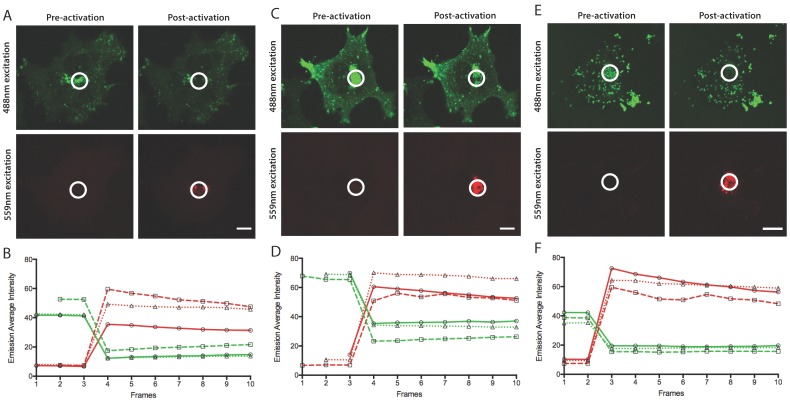
Efficiency of photoconversion of Dendra2 fusion proteins expressed by recombinant viruses. Micrographs of HeLa cells infected with (**A**) B5R-Dendra2, (**C**) F13L-Dendra2 and (**E**) Dendra2-A3L viruses at 8 hpi. The site of wrapping (**A**,**C**) or the virus factory (**E**) was identified as the most intense Dendra2 localization (pre-conversion) in infected cells. Photoconversion was performed at indicated regions of interest (circle). Excitation with 488 nm and 559 nm was used to simultaneously collect emission spectra corresponding to pre-conversion (green) and post-conversion (red) states. Scale bars are 10 μm. The pre-conversion and post-conversion emission states within the region of interest were plotted over time in three replicate cells (dotted, dashed and solid lines) for each virus **(B**,**D**,**F**). The dashed line corresponds to the cell shown in (**A**,**C**,**E**). Photoconversion was performed following frame 3 (**B**,**D**) and frame 2 (**F**).

In a B5R-Dendra2-infected cell (Figure 3A), photoconversion led to a 70.2% decrease in the emission of the pre-conversion state (green light, a 3.4-fold change) with a concurrent 533.6% increase in emission of the post-conversion state (red light, a 5.3-fold change) (Figure 3B). This indicates a 17.9-fold change (the product of the fold changes in the pre- and post-conversion emission) in the fluorescence signal between non-photoconverted and photoconverted forms of the fusion protein (replicate cells showed 24.2 and 22.5-fold changes). In the F13L-Dendra2-infected cell (Figure 3C), photoconversion led to a 49.5% decrease in the emission of the pre-conversion state (a 2.0-fold change) with a concurrent 433.2% increase in the emission of the post-conversion state (a 4.3-fold change) (Figure 3D). This indicates a 8.6-fold change in the fluorescent signal between non-photoconverted and photoconverted forms (replicate cells showed 20.6 and 13.4-fold changes). In the Dendra2-A3L-infected cell (Figure 3E), photoconversion led to a 53.7% decrease in the emission of the pre-conversion state (a 2.2-fold change) with a concurrent 708.9% increase in the emission of the post-conversion state (a 7.1-fold change) (Figure 3F). This indicates a 15.3-fold change in the fluorescent signal between non-photoconverted and photoconverted states (replicate cells showed 19.8 and 13.5-fold changes). While lower average fold changes were achieved with F13L-Dendra2- and Dendra2-A3L- infected cells, these were not indicative of less efficient photoconversion than B5R-Dendra2 but were rather due to cell-to-cell variation. Photoconversion of Dendra2 did not lead to high fold changes in the emission of the pre-conversion state compared to the observed changes in the post-conversion state. 

**Figure 4:**
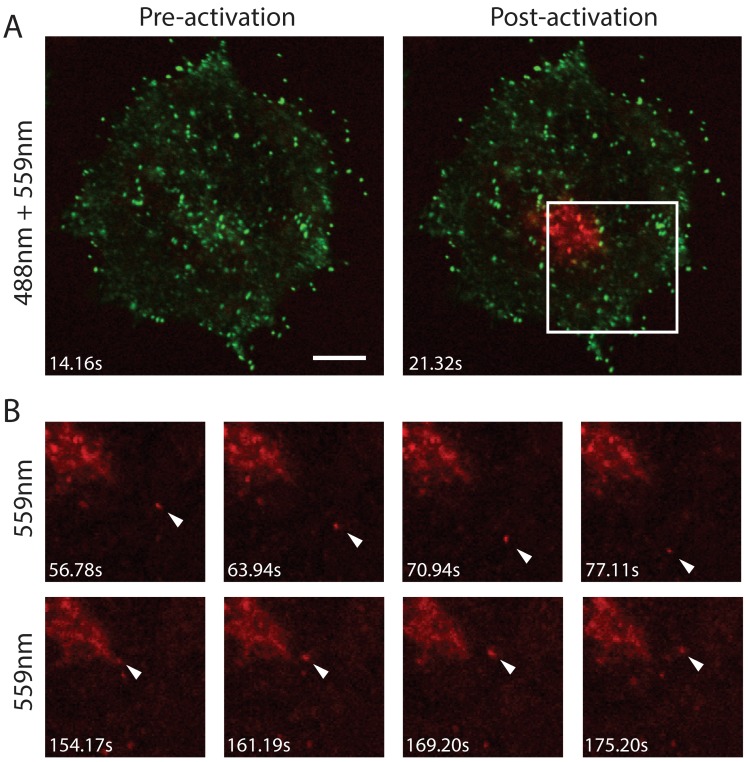
Egress of photoconverted WV from the site of wrapping. (**A**) The site of wrapping in an F13L-Dendra2 infected HeLa cell at 8 hpi was photoconverted. (**B**) The egress of two photoactivated WV (inset) was tracked over 21.67 s. Arrows indicate photoactivated WV. Scale bar is 10 μm.

The high contrast between pre-conversion and post-conversion Dendra2 emission characteristics prompted us to attempt tracking the trajectory of selected cohorts of virus particles. In cells infected at 7–9 hpi with F13L-Dendra2, the TGN was selected as a region of interest and photoconverted (Figure 4A). Following photoconversion, imaging of live cells revealed the rapid appearance of distinct virus-sized objects in the cytoplasm that displayed the excitation and emission characteristics of photoconverted Dendra2 protein (Figure 4B). These objects were subsequently observed to undergo transport in a saltatory fashion consistent with microtubule-based motility. Based on their speeds, localization and the expression of the WV-specific F13 fusion protein, these objects were assumed to be WV.

**Figure 5:**
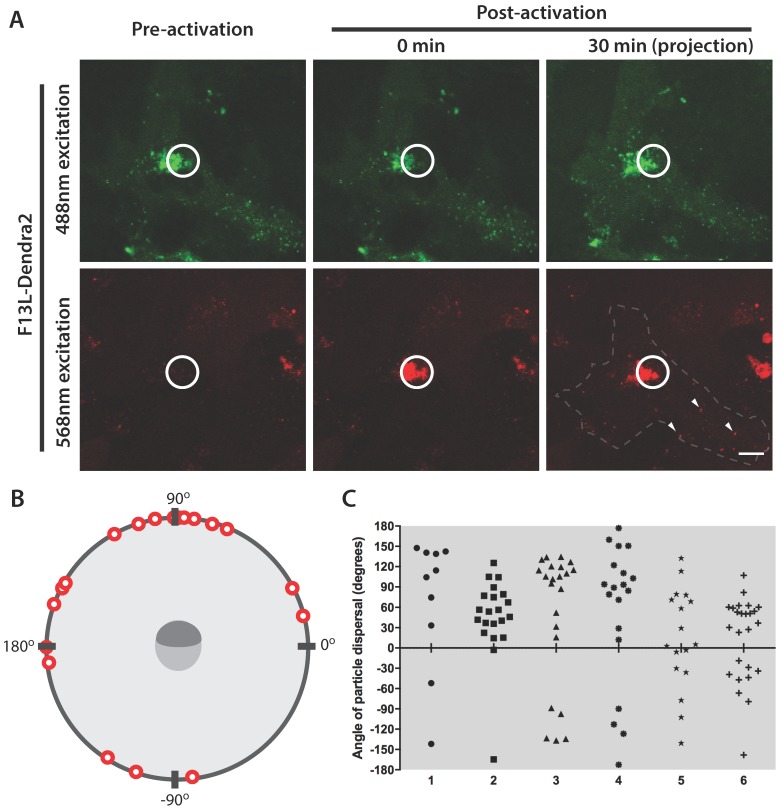
Directionality of WV egress from the site of wrapping. (**A**) Micrographs of a HeLa cell infected with F13L-Dendra2 at 8 hpi. Approximately 50% of the site of wrapping was photoconverted at the indicated region of interest (circle). At 30 min postconversion, z-stacks of the pre-conversion and post-conversion states were collected and a projection of all slices was generated. Examples of WV that have migrated out of the region of interest are indicated with arrowheads. (**B**) A schematic representation of the resultant angle of WV egress in relation to the side of the site of wrapping that was photoconverted (represented in dark grey) at 30 min postconversion from a representative cell. The directionality of WV egress from six replicate cells (**C**). The angle of egress as it corresponds to the photoconverted half of the site of wrapping is indicated in (**B**). Scale bar is 10 μm.

All WV are generated at the TGN, where MV acquire a double membrane loaded with WV-specific proteins, and then disperse throughout the cytoplasm. We wondered whether the origin of WV within TGN could predict their eventual destination at the cell periphery. To test this hypothesis, we selected a region of interest for photoconversion that encompassed one half of the TGN and imaged a z-stack at 30 min post-conversion to enumerate all the photoconverted WV within the cell (Figure 5A). Virus particles were also given a value from −180° to 180° to indicate whether they had dispersed from the same side of the photoconverted TGN or the opposite side (Figure 5B). We observed that the angle of dispersal was strongly affected by the origin within the TGN, with 59 to 90% of WV displaying a preference for the same side as the photoconverted half of the TGN (Figure 5C). This indicates that the relative presumed position to the microtubule organizing centre, based on the centrally localized F13-Dendra accumulation, strongly affects the directionality of trafficking of WV.

### 3.3. F12L and A36R Mutants Reveal a Role for Kinesin-1 in WV Exit from the TGN

**Figure 6:**
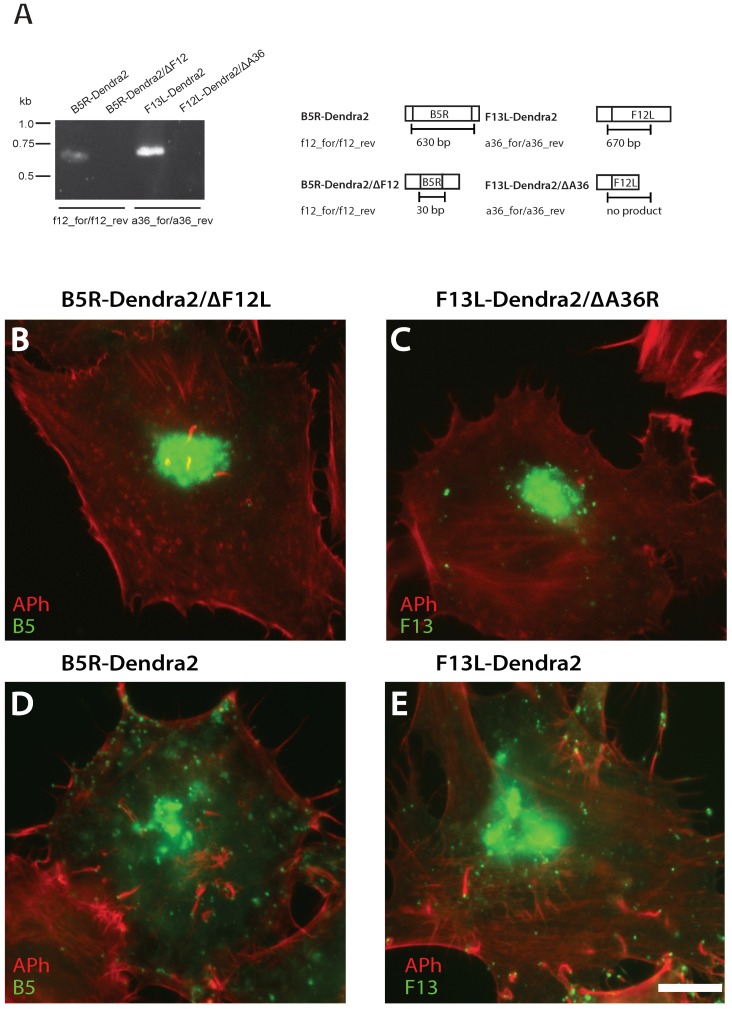
F12L and A36R PCR products analyzed by gel electrophoresis. (**A**) Genomic regions encompassing the A36R or F12L alleles were amplified by PCR and analyzed by gel electrophoresis. (**B**,**C**). Micrographs of HeLa cells infected with B5R-Dendra2/ΔF12L and F13L-Dendra2/ΔA36R viruses at 8 hpi. Cells were stained with phalloidin (red). (**D**,**E**) B5-Dendra2 and F13-Dendra2 are shown in green. Scale bar is 10 μm.

The kinesin-1 microtubule motor complex mediates anterograde transport of WV to the cell periphery and kinesin-1 recruitment to WV is affected by A36 and F12. To further characterize WV egress from the TGN and the role of kinesin-1 engagement, we introduced null alleles A36R and F12L into B5R-Dendra2 and F13L-Dendra2 backgrounds, respectively. The successful introduction of the A36R and F12L null alleles was confirmed by PCR of genomic viral DNA (Figure 6A). F13L-Dendra2 was initially selected as it gave superior WV visualization and photoconversion but we failed to recombine ΔF12L into an F13L-Dendra2 background due to the proximity of these loci or the potential non-viability of these viruses. As expected, loss of A36R and F12L resulted in a reduction in virus particle spread compared to the parental strains (Figure 6B–E).

**Figure 7:**
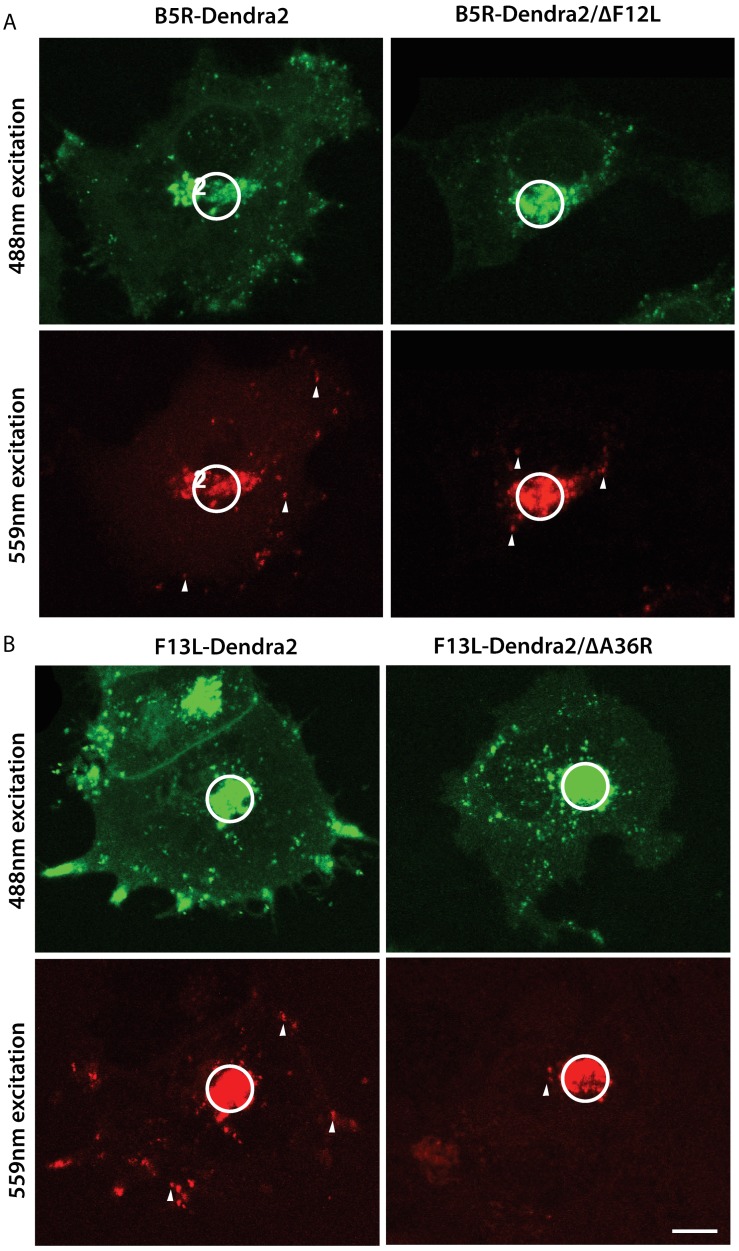
Effects of deleting A36R and F12L on WV egress from the site of wrapping. Micrographs of HeLa cells infected with (**A**) B5R-Dendra2 and B5RDendra2/ΔF12L, (**B**) F13L-Dendra2 and F13L-Dendra2/ΔA36R viruses at 8 hpi. The site of wrapping was photoconverted at the indicated region of interest (circle). At 30 min postconversion, z-stacks of the pre-conversion and post-conversion states were collected and a projection of all slices was generated. Examples of WV that egressed to the cytoplasm from the region of interest are indicated with arrowheads. Scale bar is 10 μm.

**Figure 8:**
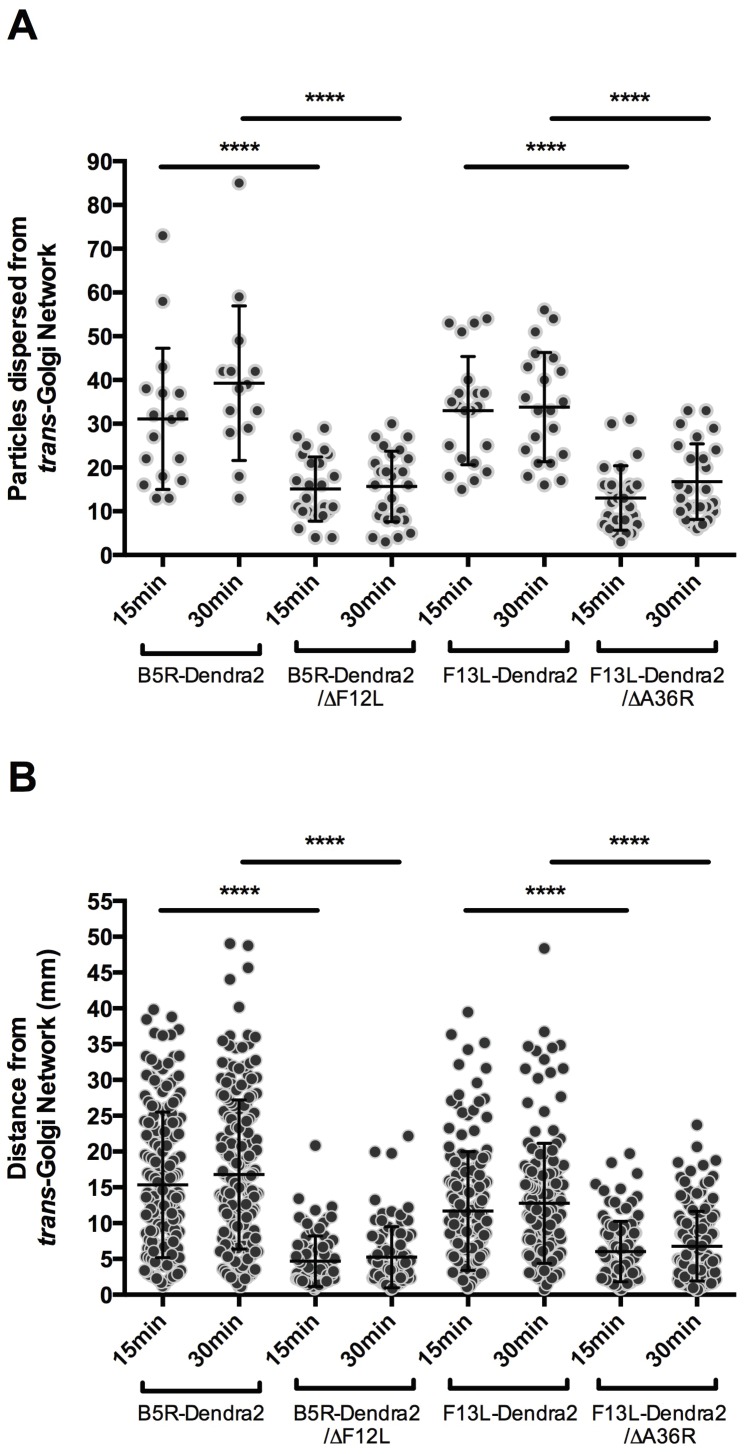
Dynamics of WV egress from the site of wrapping and the role of A36R and F12L. HeLa cells were infected with B5R-Dendra2, B5RDendra2/ΔF12L, F13L-Dendra2 and F13L-Dendra2/ΔA36R viruses and at 8 hpi the site of wrapping was photoconverted and a z-stack was collected of the pre-converted and post-converted states at 15 and 30 min postconversion. (**A**) WV that had egressed from the site of wrapping were enumerated (*n* = 18 cells, pooled from at least 3 replicates). (**B**) The distance traversed by WV from the edge of the photoconverted region was measured (6 cells for each condition, two randomly selected in each of the three replicates). WV per cell ranged from *n* = 5 to *n* = 48. Significance is indicated as follows: **** (*p* ≤ 0.0001).

To characterise the dynamics of WV transport out of the TGN, a series of live cell imaging experiments were carried out with B5R-Dendra2, B5R-Dendra2/∆F12L, F13L-Dendra2 and F13L-Dendra2/∆A36R. A region of interest encompassing the TGN was selected for photoconversion and at 15 min and 30 min post-conversion, z-stacks were collected to capture all photoconverted and non-photoconverted WV that had dispersed over that time period (Figure 7A,B). Z-projections were then used to enumerate the number of WV that had exited the TGN and the distance they had traversed in the cytoplasm (Figure 8A,B). A mean of 31.1 ± 3.92 particles were transported out of the TGN at 15 min post-activation for cells infected with B5R-Dendra2, 15.1 ± 1.47 for B5R-Dendra2/∆F12L, 33.0 ± 2.70 for F13L-Dendra2 and 13.0 ± 1.44 for F13L-Dendra2/∆A36R (Figure 8A). For all viruses, the bulk of virus dispersal from the TGN occurred in the first 15 min post photoconversion, although there is a slight increase, albeit insignificant, in the mean number of virus particles dispersed from the TGN at the 30 min time point compared to the 15 min time point. Comparisons in the number of particles dispersed in different virus infections showed that both mutations led to reductions relative to their parental strains. B5R-Dendra2/∆F12L showed a mean reduction of 16.0 virus particles from B5R-Dendra2 at 15 min post-conversion (*p* < 0.0001) and 23.6 at 30 min post-conversion (*p* < 0.0001). A similar reduction was seen between F13L-Dendra2/∆A36R and F13L-Dendra2, with a 20.0 difference at 15 min (*p* < 0.0001) and 17.0 difference at 30 min post-conversion (*p* < 0.0001).

To further elucidate differences between our parental and ∆A36R and ∆F12L strains, we performed a second set of analyses on these same image series, measuring the distance travelled by virus particles. B5R-Dendra2 particles were transported a mean distance of 15.3 ± 0.78 µm from the TGN at 15 min post-conversion, B5R-Dendra2/∆F12L were transported 4.7 ± 0.38 µm, F13L-Dendra2 were transported 11.7 ± 0.64 µm and F13L-Dendra2/∆A36R were transported 6.0 ± 0.41 µm (Figure 8B). Similar to the number of particles dispersed out of the TGN, the majority of the movement had occurred by the 15 min post-conversion time point, although there is a slight increase in the mean of distance dispersed at the 30 min time point compared to the 15 min time point. Comparisons in the distances virus particles were transported between parental and ∆A36R and ∆F12L viruses showed that, as expected, WV transport was strongly attenuated in strains where kinesin-1 engagement was disrupted. 

## 4. Discussion

Here we achieved efficient photoconversion of the site of wrapping (TGN) in cells infected with recombinant VACV expressing PAFP-fusion proteins. This allowed us to track virus particle trajectories of a subset of the virus particle population within a cell. Dendra2 proved to be a superior PAFP for the purposes of the live-cell imaging of virus movement owing to the high efficiency of photoconversion and other spectral characteristics such as low rates of background photoconversion, photostability and brightness. In our preliminary studies, we generated VACV carrying B5 and A3 fusions to PS-CFP2 (data not shown). Our results with these strains revealed that viral particles were inefficiently photoconverted and were unable to be resolved at high resolution in their pre-photoconversion states due to low brightness. The PS-CFP2-A3L did form sufficiently bright particles in the pre-conversion state due to the abundance of A3 localized to the core but imaging of the post-conversion state uses the same wavelength (405 nm) as that required for photoconversion. Imaging post-conversion infected cells led to inadvertent photoconversion even at the lowest laser intensity, a problem exacerbated by sequential imaging. These issues were not encountered with Dendra2-expressing VACVs in which we achieved changes in emission intensity of between 10- and 25-fold. Although this figure is well below the reported theoretical achievable fold-change for Dendra2 of 4000-fold [5], it is comparable to other studies; for example tracking the movement of Dendra2-tagged transcription factors through the *Arabidopsis* root (10-fold, [36]) and the original study describing the improved Dendra2 mutant (10-fold, [5]).

Other uses of photoconvertible fluorescent proteins in viral contexts have included tracking HIV-1 Pr55-Gag, the precursor for all structural determinants for HIV-1 particle formation (tagged with PA-GFP [37]) and tracking NS5A, a key protein in the replication of Hepatitis C RNA replication (tagged with PA-GFP [38]). These studies have aimed to monitor the motility of proteins over the course of viral assembly and replication, rather than marking virus particles to analyze their subcellular transport. In another study, HIV genomes were labeled by the expression of Eos fusion protein that is able to bind full-length engineered HIV RNA molecules [39]. This approach was used to characterise the turnover of viral RNAs in the vicinity of the plasma membrane by specifically photoconverting Eos with a near-UV laser at a TIRF angle. The region of interest was restricted to the *Z*-axis rather than the *X*-*Y* axes as in our experiments.

We used our photoconvertible VACV strains to examine dispersal of WV from the TGN, a phenomenon difficult to study with viruses labeled with standard fluorescent proteins due to the abundance of virus particles that appear in the cytoplasm. We observed that the bulk of photoconverted WV exit from the TGN occurs in the first 15 min post photoconversion, with a slight, though statistically insignificant, increase in the mean of virus particle number dispersed from the TGN at the 30 min time point. Our interpretation of these data is that at 15 min the WV in the cytoplasm have almost reached equilibrium with newly egressed WV being compensated by WV that have been released from the cell. Judging from the fluorescence of the TGN, it does not appear to be exhausted of photoconverted Dendra2 protein.

As previously described, both A36 and F12 (and E2, which complexes with F12) have been implicated in microtubule-based transport, with deletion mutants displaying defective egress of WV [13,17,20]. We demonstrated that the number of particles transported out of the TGN at the 15 min and 30 min time points are not significantly different to each other for each of the two parental strains B5R-Dendra2 and F13L-Dendra2 (*p* = 0.67 and *p* = 0.29, respectively) and likewise that there were not significant differences between each time point for either of the two mutant viruses (*p* = 0.33 and *p* = 0.64). It has been previously demonstrated that fewer Cell-associated Enveloped Virus (CEV) are formed in a ∆F12L background than a ∆A36R background [20]. Our data support the hypothesis that this is due to WV particles in a ∆F12L background having shorter run lengths in the cytoplasm than ∆A36, as opposed to fewer WV particles exiting the TGN. This disparity may suggest a difference in the affinity or recruitment of ∆F12L and ∆A36R viral cargoes to microtubule motors. While these data indicate that loss of F12 has a more profound impact on virus egress than loss of A36, the observed difference in virus egress may result from synergistic interactions between the fusion of Dendra2 to B5R or F13L and the loss of F12 or A36 function creating a more profound impact on virus egress than if any of these mutations were present individually. Indeed, this has been shown to occur with some combinations of fluorescent protein to envelope protein fusions and envelope protein deletions [21]. Many viruses, including PRV [40,41], HSV [42,43] and HIV-1 [44] have been visualized undergoing saltatory bidirectional movement despite exhibiting overall anterograde progress. This bidirectionality may play roles in important processes such as navigating around physical blockades along particular microtubule paths [43,45]. In HSV, this process involves the simultaneous binding of motors with opposing directionalities (kinesin-1 and dynein) that are regulated to achieve effective transport to the cell periphery [46,47]. While it is possible that there is a similar process occurring in VACV egress, kinesin-1 at least appears to play a dominant role in the egress of VACV WV and subsequent attenuation of ∆F12L and ∆A36R deletion mutants. As both A36 and F12 proteins are able to interact with kinesin-1, the variable run length of deletion viruses may be due to the differential recruitment of KLC isoforms to the virus [16,18]. Our results support a role for kinesin-1 in WV exit from the TGN, a function disrupted with loss of either A36 or F12.

## Figures and Tables

**Table 1:** Primers for plasmid constructs.

Name	Sequence
a3_1	AAGTCGACCGTTGACGCCGAGCAATGC
a3_2	TTGGATCCCGAGAATGAATAAGTACTAAAGG
a3_3	AAGGCGGCCGCGAAGCCGTGGTCAATAGCG
a3_4	TTAAGCTTCGAGAATGAATAAGTACTAAAGG
a3_5	AAAGATCTGTCGACCGTTGACGCCGAGCAATGC
a3_6	GTTAATTCCCGGGGTGTTCATTATTTATATTCGTAGTTTTTAC
a3_7	GTAAAAACTACGAATATAAATAATGAACACCCCGGGAATTAAC
den_1	TTGCGGCCGCCCCACACCTGGCTGGGCAGGGG
den_2	AAAGATCTACCATGAACACCCCGGGAATTAAC
den_3	AAGCGGCCGCCAACACCCCGGGAATTAACCTG
den_4	TTGGATCCTTACCACACCTGGCTGGGCAG
b5_1	GTTCCATAAATTGCTACCG
b5_2	AAAGGATCCTATACCATTAAGTGTATCCATCACC
f13_1	AAAGATCTACCATGTGGCCATTTGCATCG
f13_2	TTGCGGCCGCCAATTTTTAACGATTTACTGTG

**Table 2:** Primers for viral genomic DNA.

Name	Sequence
f12_for	GAATATCCTGCTCTGATAGCAG
f12_rev	GCTAGGGTTATTTGGATGGATGCG
a36_for	ATGATGCTGGTACCTATCACG
a36_rev	CACGAACAGGGAGATATAGCAC
